# Silencing of *Opisthorchis viverrini* Tetraspanin Gene Expression Results in Reduced Secretion of Extracellular Vesicles

**DOI:** 10.3389/fcimb.2022.827521

**Published:** 2022-02-11

**Authors:** Sujittra Chaiyadet, Javier Sotillo, Watchara Krueajampa, Sophita Thongsen, Michael Smout, Paul J. Brindley, Thewarach Laha, Alex Loukas

**Affiliations:** ^1^ Tropical Medicine Graduate Program, Academic Affairs, Faculty of Medicine, Khon Kaen University, Khon Kaen, Thailand; ^2^ Parasitology Reference and Research Laboratory, National Centre for Microbiology, Instituto de Salud Carlos III, Majadahonda, Madrid, Spain; ^3^ Department of Parasitology, Faculty of Medicine, Khon Kaen University, Khon Kaen, Thailand; ^4^ Centre for Molecular Therapeutics, Australian Institute of Tropical Health and Medicine, James Cook University, Cairns, QLD, Australia; ^5^ Department of Microbiology, Immunology and Tropical Medicine, and Research Center for Neglected Diseases of Poverty, George Washington University, Washington, DC, United States

**Keywords:** *Opisthorchis viverrini*, liver fluke, extracellular vesicle, endocytosis, tetraspanin

## Abstract

Inter-phylum transfer of molecular information is exquisitely exemplified in the uptake of parasite extracellular vesicles (EVs) by their target mammalian host tissues. The oriental liver fluke, *Opisthorchis viverrini* is the major cause of bile duct cancer in people in Southeast Asia. A major mechanism by which *O. viverrini* promotes cancer is through the secretion of excretory/secretory products which contain extracellular vesicles (*Ov*EVs). *Ov*EVs contain microRNAs that are predicted to impact various mammalian cell proliferation pathways, and are internalized by cholangiocytes that line the bile ducts. Upon uptake, *Ov*EVs drive relentless proliferation of cholangiocytes and promote a tumorigenic environment, but the underlying mechanisms of this process are unknown. Moreover, purification and characterization methods for helminth EVs in general are ill defined. We therefore compared different purification methods for *Ov*EVs and characterized the sub-vesicular compartment proteomes. Two CD63-like tetraspanins (*Ov*-TSP-2 and TSP-3) are abundant on the surface of *Ov*EVs, and could serve as biomarkers for these parasite vesicles. Anti-TSP-2 and -TSP-3 IgG, as well as different endocytosis pathway inhibitors significantly reduced *Ov*EV uptake and subsequent proliferation of cholangiocytes *in vitro*. Silencing of *Ov-tsp-2* and *tsp-*3 gene expression in adult flukes using RNA interference resulted in substantial reductions in *Ov*EV secretion, and those vesicles that were secreted were deficient in their respective TSP proteins. Our findings shed light on the importance of tetraspanins in fluke EV biogenesis and/or stability, and provide a conceivable mechanism for the efficacy of anti-tetraspanin subunit vaccines against a range of parasitic helminth infections.

## Introduction

Perhaps the best example of inter-kingdom molecular communication is that between parasitic helminth-derived extracellular vesicles (EVs) and the cells that they target lining the epithelial/endothelial surfaces of their respective vertebrate hosts. For example, the carcinogenic liver fluke, *Opisthorchis viverrini*, resides in the bile ducts of its human host where it secretes EVs that are internalized by biliary epithelial cells, or cholangiocytes (Chaiyadet et al., 2015b). Over 8 million people are infected in Thailand with *O. viverrini* (Sripa et al., 2007), and chronic *O. viverrini* infection has been strongly linked to cholangiocarcinoma, a form of bile duct cancer which has the highest global prevalence in the Northeast region of Thailand (Sripa and Pairojkul, 2008). Multiple processes are involved in liver fluke-induced cancer, including mechanical damage caused by the parasite’s physical attachment to and grazing on the biliary epithelium, chronic immunopathological processes that induce pro-inflammatory cytokines such as IL-6, and the release of parasite-derived excretory/secretory (ES) products into the bile duct that drive unchecked cell proliferation (Sripa et al., 2018). ES products are highly immunogenic (Wongratanacheewin et al., 1988; Sripa and Kaewkes, 2000a; Sripa and Kaewkes, 2000b; Choi et al., 2003) and contain dozens of soluble proteins and other material such as extracellular vesicles (EVs) that promote a variety of changes in the host (Mulvenna et al., 2010; Chaiyadet et al., 2015b; Suttiprapa et al., 2018).

EVs can be subdivided into different categories based mainly on size and composition. Smaller EVs (traditionally termed exosomes) pellet at 120,000 *g*, have a size of 30-120 nm and are formed within multivesicular bodies (MVBs); larger vesicles >200 nm in size, also known as 15,000 *g* EVs or microvesicles, are formed by direct budding of the plasma membrane of cells (Doyle and Wang, 2019). Numerous studies have established the roles of defined EV surface proteins in the interactions between vesicles and their target cells, including ICAM-1 (Segura et al., 2005), integrins and the tetraspanins (TSPs) CD9 and CD63 (Morelli et al., 2004). Several mechanisms by which EVs deliver their content into target cells have been described (Mulcahy et al., 2014; Mathieu et al., 2019), including clathrin-dependent mechanisms in neurons (Fruhbeis et al., 2013) and clathrin-independent mechanisms in endothelial cells (Nanbo et al., 2013; Svensson et al., 2013), or even fusion of the EV membrane with the plasma membrane of its target cell (Parolini et al., 2009; Montecalvo et al., 2012; Andreu and Yanez-Mo, 2014).

EVs have emerged as a ubiquitous mechanism for mediating inter-kingdom and inter-phylum communication between parasites and their hosts (Wu et al., 2018). Indeed, in recent years a number of studies have analyzed the molecular composition of liver fluke EVs and their impact on host cell lines *in vitro* (Bernal et al., 2014; Chaiyadet et al., 2015b; Cwiklinski et al., 2015). We characterized the proteome of *O. viverrini* EVs (*Ov*EVs) and documented their internalization *in vitro* by a human cholangiocyte cell line, and demonstrated subsequent proliferation and release of pro-inflammatory cytokines such as IL-6 by these cells (Chaiyadet et al., 2015b). However, the exact mechanisms by which helminth EVs interact with their host cells is still unclear.

Tetraspanins (TSPs) are a family of proteins consisting of four conserved transmembrane domains, a small extracellular loop (Thery et al., 2018) and a large extracellular loop (LEL) (Kitadokoro et al., 2001). In general, they are involved in many biological processes (Termini and Gillette, 2017), and in helminths they have been associated with the development, maturation and structural integrity of the tegument (Piratae et al., 2012; McWilliam et al., 2014; Chaiyadet et al., 2017). TSPs are abundant on the surface of EVs and are widely considered as a bona fide marker of mammalian exosomes. TSPs play an important role in EV-cell interactions, and blocking of CD81 and CD9 results in the inhibition of the internalization process (Nazarenko et al., 2010; Rana et al., 2012; Zech et al., 2012). In terms of helminths, we have already demonstrated that antibodies against *O viverrini* TSP-1 (CD9-like) and TSP-2 (CD63-like) blocked internalization of *Ov*EVs by cholangiocytes (Chaiyadet et al., 2015b; Chaiyadet et al., 2019), and more recently it was shown that antibodies against the CD63-like *Sm*-TSP-2 on the surface of EVs from the related fluke, *Schistosoma mansoni* also blocked their uptake by vascular endothelial cells and monocytes (Kifle et al., 2020a).

In the current study we present the sub-vesicular proteomes of *O. viverrini* EVs following a more comprehensive purification and isolation method than previously reported for fluke EVs, and analyze the mechanisms involved in EV uptake by host cells, including the importance of TSPs in extracellular vesicle secretion.

## Materials and Methods

### Animal Ethics

Male Syrian golden hamsters 6-8 weeks were infected with metacercariae, maintained for 8 weeks, and euthanised for worm collection as described elsewhere (Sripa and Kaewkes, 2000b). Animals were maintained in the animal care unit at the Faculty of Medicine, Khon Kaen University. Animal experiments were approved by Animal Ethics Committee of Khon Kaen University (ACUC KKU 31/2561). New Zealand white rabbits were maintained in the Northeast Laboratory Animal Center, Khon Kaen University, where they were vaccinated with recombinant TSP proteins to raise antibodies (Chaiyadet et al., 2017).

### Isolation of Excretory/Secretory Products and Purification of Extracellular Vesicles

Adult worms were collected from hamster livers 8 weeks post-infection, washed in PBS and cultured in RPMI 1640 containing 1% glucose and 100 units/ml Penicillin and 100 units/ml Streptomycin (Life Technologies, Grand Island, NY) and 1 nM E64 (Thermo scientific, USA) at 37°C, 5% CO_2_ for 7 days. Culture media containing the *O. viverrini* ES products (*Ov*ES) was collected every day, centrifuged at 500 g for 10 min to remove eggs and large debris, and subsequently centrifugated at 2,000 *g* for 30 min, 4,000 *g* for 30 min, and 12,000 *g* for 45 min to remove smaller debris and MVs. Supernatant was collected, concentrated using a 10 kDa cut-off Amicon filter (Merk Millipore, USA) and ultracentrifuged at 120,000 *g* for 3 h. The pellet was dissolved in PBS, then centrifuged again at 120,000 *g* for 3 h. The pellet was either analyzed immediately using tunable resistive pulse sensing (TRPS) as described below or collected and dissolved in 70 µl of PBS to perform further purification using iodixanol by laying the pellet on a discontinuous gradient (40%, 20%, 10%, 5%) built with OptiPrep™ Density Gradient (Sigma, USA) as described previously (Sotillo et al., 2016). A total of 12 fractions were obtained.

### Analysis of Extracellular Vesicles Using Tunable Resistive Pulse Sensing

Vesicle protein content was determined by BCA (Bio-Rad). The particle concentration and size distribution of each fraction collected after (i) ultracentrifugation only and (ii) iodixanol gradient was determined by TRPS using a qNano instrument (Izon Science, New Zealand) following the manufacturer’s instructions. Briefly, a nanopore NP150 was used, and pressure and voltage values were set to optimize the signal to ensure high sensitivity. Samples were diluted 1:5 in electrolyte (Izon Science) before applying to the nanopore. Calibration was performed using CP100 carboxylated polystyrene calibration particles (Izon Science) at a 1:2,000 dilution. Size distribution and concentration of particles were analyzed using the software provided by Izon (version 3.2), and purity analysis (number of particles/µg of protein) was performed following established methods (Webber and Clayton, 2013).

### Proteomic Analysis of *O. viverrini* 120k EVs

EV surface-exposed peptides were released by trypsin hydrolysis following established methodology with modifications (Cwiklinski et al., 2015). Briefly, surface-exposed proteins were cleaved by trypsin (1 µg/µl) for 15 min at 37°C. Treated EVs were then centrifuged at 120,000 *g* for 1 h at 4°C, and supernatant containing the surface peptides collected. The pellet was further processed to investigate proteins associated with the EVs membrane and within the EV lumen. Briefly, EVs were resuspended in water, sonicated and released EV cargo content was recovered from the supernatant after centrifugation at 120,000 *g* for 1 h at 4°C. Subsequently, the pellet was solubilized in 1% Triton X-100/2% sodium dodecyl sulphate (SDS) at 37°C for 15 min to recover membrane-associated proteins. For the proteomic analysis, cargo and membrane-associated proteins were electrophoresed on a 10% SDS-PAGE gel for 2-3 cm to remove detergent and salt from the sample prior to reduction and alkylation. In-gel trypsin digestion was performed as described previously with minor modifications (Sotillo et al., 2014). Briefly, the gel was stained with Coomassie blue (0.03%), destained and each lane was cut into 5 slices. Each band was further destained in 50% acetonitrile with 200 mM ammonium bicarbonate (Sigma-Aldrich, USA) at 37°C for 45 min, reduced with 20 mM dithiothreitol (Sigma-Aldrich, USA) in 25 mM ammonium bicarbonate for 1 h at 65°C, alkylated with 50 mM iodoacetamide (Sigma-Aldrich, USA) in 25 mM ammonium bicarbonate at 37°C for 15 min and digested with 100 ng/µl trypsin (Sigma-Aldrich, USA) at 37°C overnight. To maximize peptide recovery, gel pieces were further incubated in 50% acetonitrile with 0.1% trifluoroacetic acid and supernatant was combined for each slice. All peptides were finally dried in a vacuum concentrator. Finally, samples were desalted using a Zip-Tip^®^ column (Merck Millipore) pipette tip according to the manufacturer’s protocol and dried in a vacuum concentrator before analysis using liquid chromatography-tandem mass spectrometry (LC-MS/MS).

### LC-MS/MS Analysis and Database Search

All samples (trypsin-liberated, cargo and membrane proteins) were injected into an Eksigent nanoLC 415 for peptide separation using a linear gradient and eluates were directly introduced into the PicoView ESI ionisation source of a TripleTOF 6600 MS/MS System (AB Sciex) as described previously (Kifle et al., 2020b).

Peak lists obtained from MS/MS spectra were identified using X!Tandem version X! Tandem Vengeance (2015.12.15.2) (Craig and Beavis, 2004), MS-GF+ version Release (v2018.04.09) (Kim and Pevzner, 2014) and Tide (Diament and Noble, 2011). The search was conducted using SearchGUI version 3.3.13 (Vaudel et al., 2011). Protein identification was conducted against a concatenated target/decoy version of the *O. viverrini* genome and the common repository of adventitious proteins (cRAP database) (10,876 (target) sequences). The identification settings were as follows: Trypsin, Specific, with a maximum of 2 missed cleavages, 20.0 ppm as MS1 and 0.2 Da as MS2 tolerances; fixed modifications: Carbamidomethylation of C, variable modifications: Deamidation of N and Q, Oxidation of M (+15.994915 Da), fixed modifications during refinement procedure: Carbamidomethylation of C, variable modifications during refinement procedure: Acetylation of protein N-term, Pyrolidone from E, Pyrolidone from Q, Pyrolidone from carbamidomethylated C.

Peptides and proteins were inferred from the spectrum identification results using PeptideShaker version 1.16.40 (Vaudel et al., 2015). Peptide Spectrum Matches (PSMs), peptides and proteins were validated at a 1.0% False Discovery Rate (FDR) estimated using the decoy hit distribution. The mass spectrometry proteomics data have been deposited to the ProteomeXchange Consortium *via* the PRIDE (Perez-Riverol et al., 2019) partner repository with the dataset identifier PXD020345 and 10.6019/PXD020345.

Blast2GO software (Conesa et al., 2005) was employed for the Gene Ontology (GO) analysis. Only children GO terms were used for subsequent analysis to avoid redundancy in GO terms. Protein family (Pfam) analysis was performed using the gathering bit score (–cut_ga) threshold using HMMER v3 (Potter et al., 2018).

### Western Blotting

An equal quantity of protein from all twelve fractions of EVs obtained after iodixanol gradient, as well as the EVs collected after ultracentrifugation but prior to iodixanol gradient separation were electrophoresed on a 15% SDS-PAGE gel, and samples were transferred to nitrocellulose membrane in a transblot unit (GE healthcare). The membrane was washed with PBS-Tween 0.05% (PBST) containing 5% skimmed milk for 2 h at room temperature and probed with rabbit anti-*Ov*-TSP-2 and anti-*Ov*-TSP-3 at 1:300 dilution overnight at 4°C, followed by goat anti-rabbit-HRP diluted 1:1,000 in PBST (Merk Millipore, USA). Membranes were developed with Luminata™ Forte Western HRP Substrate (Merk Millipore, USA) and images were captured using an imageQuant LAS 500 chemiluninescence CCD camera (GE Healthcare life Sciences, USA). To detect expression of *Ov*-TSP-2 and TSP-3 in the 120k EVs from double-stranded (ds) RNA-treated worms, EVs were isolated from the ES products of worms cultured for one or three days, and a total of 2 µg of EV total protein was electrophoresed and immunoblotted as described above. Densitometry was used to quantify the amount of protein detected by western blotting using imageJ software 1.52p (http://imagej.nih.gov/ij) and the relative level of protein expression was normalized to the mock control using the formula: Intensity of the experimental sample/Intensity of mock control sample.

### Synthesis of Double-Stranded RNAs and Electroporation of Flukes

dsRNAs of *Ov-tsp-2, Ov-tsp-3*, and firefly luciferase target loci were constructed from plasmids using primers flanked with a T7 RNA polymerase promoter sequence at the 5′ end (**Supplementary Table S1**) using a MEGA script RNAi kit (Amicon, USA), following the manufacturer’s instructions as previously described (Chaiyadet et al., 2017). Adult worms were washed with PBS three times prior to electroporation. For dsRNA electroporation, 30 worms in each group were placed in 500 µl of culture media (RPMI supplemented with 1% glucose, 1 µm E64, 1X Penicillin-Streptomycin) containing 50 µg *Ov-tsp-2*, *Ov-tsp-3* or *luc* dsRNAs in a 4 mm gap electroporation cuvette (Bio-Rad, Hercules, CA, USA). The samples were single pulsed by square wave electroporation at 125 V, 20 ms duration (Electroporation Gene Pulser Xcell, Bio-Rad). After pulsing, worms were left soaking with 2 µg dsRNAs in RPMI supplemented with 1% glucose at 37°C with 5% CO_2_ atmosphere for up to 7 days with changes of media and dsRNAs every second day. The culture media from dsRNA-treated worms was collected to purify EVs on days 1 and 3, and EVs were collected as described above.

### Cell Culture

H69 human cholangiocytes were cultured in Dulbecco’s modified Eagle’s medium (DMEM)/Ham-F12 (Gibco, USA) containing 10% fetal bovine serum (FBS), 100 Units/ml Penicillin and 100 Units/ml Streptomycin (Life Technologies, USA) and supplemented with insulin, adenine, epinephrine, T3-T, epidermal growth factors (EGF) and hydrocortisone (Ninlawan et al., 2010).

### Blocking Cellular Uptake of EVs Using Endocytosis Inhibitors and Antibodies Against Ov-TSP-2 and Ov-TSP-3

*O. viverrini* 120k EVs were labeled with PKH67 staining following the manufacturer’s protocol (Sigma Aldrich, USA). Briefly, 1.25 µg/ml of *Ov*EVs protein was mixed with PKH67 dye in dilution buffer C for 5 min at room temperature. Excess dye was quenched by incubation with 1% BSA for 1 minute and the unbound dye was removed using a 100 kDa cut-off Amicon filter (Merk Millipore, USA) and buffer exchanged with PBS. To observe the effect of different endocytosis inhibitors on the uptake of *O. viverrini* 120k EVs, cholangiocytes (H69) were pre-treated with one of the following: 5 µg/ml chlorpromazine (CPZ), 1 nM bafilomycin A1 or 0.3 M sucrose for 30 min at 37°C. Subsequently, the PKH67-labeled EVs were added to cholangiocytes in culture, at a final concentration of 0.3 µg EVs protein per milliliter of culture media and incubated for 2 hours at 37°C with 5% CO_2_. Otherwise, the PKH67-labeled EVs were treated with 100 mM Proteinase K with rotation for 1 h at room temperature, and then added to incubate with cholangiocyte for 2 h. To test the effect of anti-*Ov*-TSP-2 and anti-*Ov*-TSP3 IgG in the EV uptake process by cholangiocytes, *O. viverrini* 120k EVs were incubated with rabbit serum IgG antibodies at a 1:2.5 and 1:10 dilution (for rabbit anti-*Ov*-TSP-2 and rabbit anti-*Ov*-TSP-3, respectively) for 1 h at room temperature with gentle rotation, and subsequently cultured with H69 cholangiocytes for 2 hours at 37°C with 5% CO_2_.

### Confocal Microscopy

Cells were fixed with 4% paraformaldehyde and incubated for 30 min at room temperature. After washing with PBS, glycine (100 mM final concentration) was added and incubated for 15 min at room temperature. Nuclei were stained by Hoechst (Abcam, USA). Images were captured using a laser scanning confocal microscope (LSM800, Leica, USA), and the fluorescence intensity of PKH-67-stained EVs was quantified using ImageJ 1.50i. Thirty cells from 3 random microscope fields for 2 independent replicates were analyzed, and a one-way ANOVA followed by Tukey-Kramer *post-hoc* multiple comparison was performed to determine significance.

### Cholangiocyte Proliferation

The effects of various endocytosis inhibitors and antibodies on cell proliferation was analyzed by real-time monitoring cell growth using an xCELLigence system (Roche, USA). Briefly, cells were seeded (2,000 cells/well) in an E plate (Roche, USA) for 24 h. Cells were pre-treated with the clathrin-mediated endocytosis inhibitors CPZ (5 µg/ml final concentration) or 0.3 M sucrose, or the vacuolar ATPase inhibitor of endocytosis, bafilomycin A1 (1 nM final concentration) for 30 min, and subsequently, EVs were added to a final concentration of 0.3 µg/ml EV proteins and incubated for 24 h. Cells treated with EVs in the absence of any exogenous inhibitors were used as a control. Real-time cell growth was measured as cell index (CI) as previously described (Xing et al., 2005).

### Transmission Electron Microscopy

Transmission electron microscopy (TEM) was performed on *O. viverrini* 120k EVs. A total of 5 µg of *Ov*-exosomes was placed on a formvar-carbon coated EM grid (Ted Pella INC., USA) and incubated for 1 hour. The grids were washed with PBS for 5 min and samples were subsequently fixed with 100 µl of 2% paraformaldehyde for 10 min, followed by a wash with 100 µl of PBS for 3 min, 3 times. The grids were washed with 50 mM glycine/PBS for 3 min, 3 times and were blocked with 1% BSA/PBS for 10 min. Rabbit anti-*Ov-*TSP-2 or anti-*Ov-*TSP-3 sera were diluted 1:10 in 1% BSA/PBS, added to the grids and incubated for 1 h. Grids were washed three times with PBS for 3 min followed by addition of 5-10 µl of protein-A gold (Ted Pella INC., USA) diluted 1:100 in 0.1% BSA/PBS for 1 h. The grids were washed with 100 µl of PBS for 2 min, 5 times followed by fixation in 50 µl of 1% glutaraldehyde (Sigma-Aldrich, USA) for 10 min and finally washed in 100 µl of deionized water for 2 min, 5 times. EVs were contrasted with 2.5% uranyl acetate for 10 min. Samples were embedded in 0.13% methyl cellulose and 0.4% uranyl acetate for 10 min and grids were air dried for 5-10 min. The carbon coated grids were observed under a JEOL transmission electron microscope (JEM1010, Tokyo, Japan) equipped with a digital camera at 100 kV at 100,000x magnification.

### Statistical Analysis

Cell proliferation and internalization data were expressed as the mean ± standard error of two independent experiments using Graphpad Prism Software Version 6.03 (www.graphpad.com). The statistical significance was evaluated by One-Way ANOVA with Tukey-Kramer *post-hoc* multiple comparison test.

## Results

### Purification of *O. viverrini* 120k EVs Yields Highly Pure Vesicles

**Figure 1:**
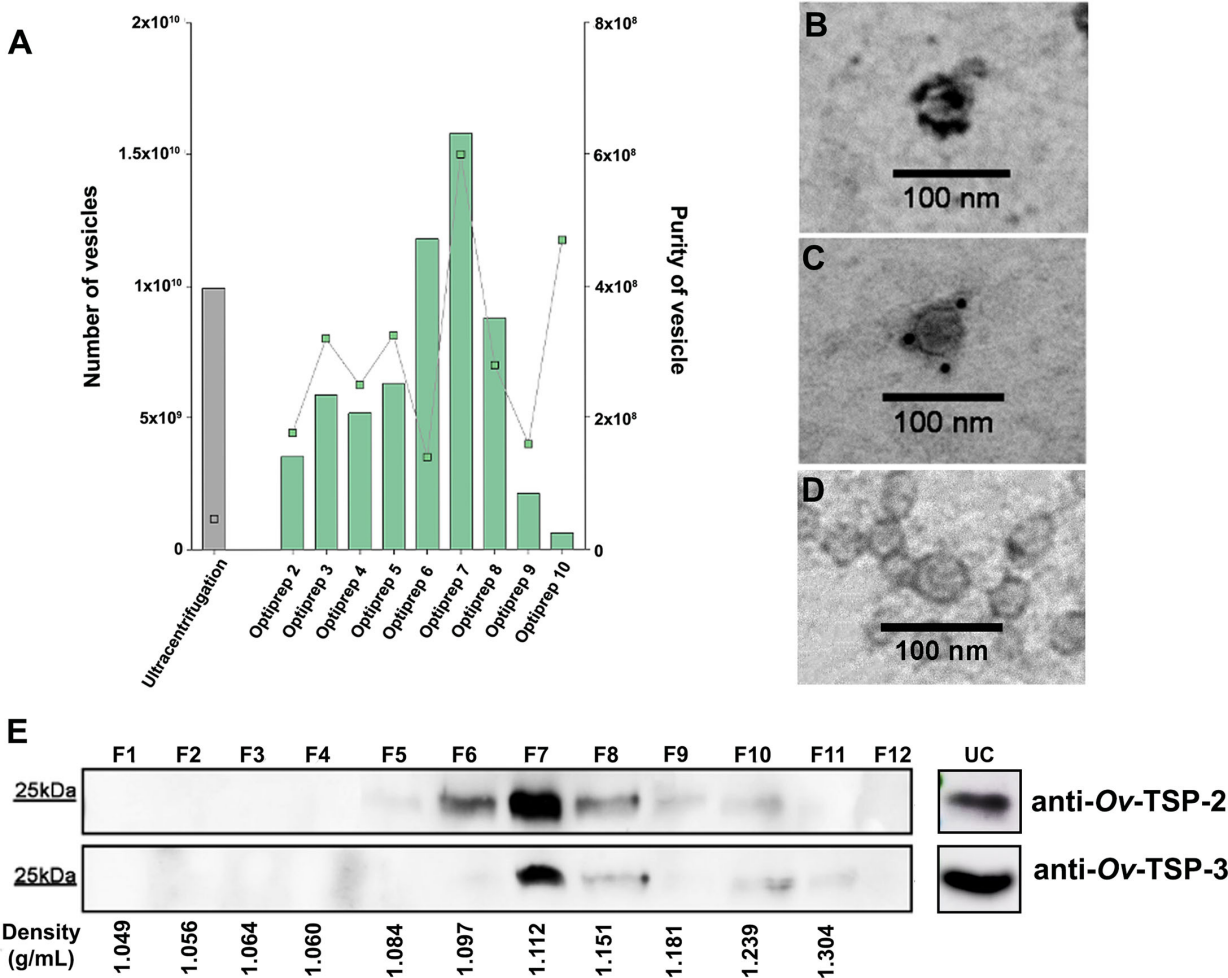
Characterisation of *Opisthorchis viverrini* 120k extracellular vesicles. *Opisthorchis viverrini* 120k extracellular vesicles (EVs) EVs were purified by Optiprep density gradient and quantified by tunable resistive pulse sensing using a qNano instrument **(A)**. Characterization of 120k EVs by immunogold transmission electron microscopy (magnification ×100,000) using rabbit anti *Ov*-TSP-2 **(B)**, anti *Ov*-TSP-3 **(C)** and normal rabbit serum **(D)** revealed the presence of both proteins on the EVs surface. An equal quantity of protein from the twelve fractions from the Optiprep density gradient of *O. viverrini* 120k EVs, as well as the ultracentrifugation fraction (prior to Optiprep gradient) were probed by western blot with the same antisera used in panels **(B, C, E)**.

To investigate the purity of EVs isolated following two standard methodologies -ultracentrifugation or ultracentrifugation coupled with iodixanol (Optiprep) density gradient, we compared the number of vesicles, purity and presence of traditional markers of mammalian cell EVs, such as tetraspanins, in all isolated fractions. The size and concentration of EVs from the two fractionation methods (including all twelve fractions obtained after iodixanol gradient) were further characterized by TRPS using a qNano instrument. The mean size of the EVs present in all samples was similar between the ultracentrifugation sample (123 ± 28 nm) and fractions F6-F9 from the iodixanol gradient sample (123 ± 23.6 - 132 ± 26 nm) (**Supplementary Table S2**). While the concentration of EVs obtained from ultracentrifugation (9.94×10^9^/ml) was similar to that obtained in most of the fractions obtained from the iodixanol gradient (6.29×10^8^/ml - 1.58×10^10^/ml), the purity of the ultracentrifugation sample was significantly lower (4.7×10^7^/ml), especially when compared to fractions F6-F9 (1.40×10^8^/ml – 6.00×10^8^/ml) (**Figure 1A**). Optiprep fractions F6-F9 had a density of 1.103-1.184g/ml, which corresponded to fractions 7-9 from the western blot analysis (**Figure 1A** and **Supplementary Table S2**).

To validate the presence of tetraspanin proteins on the 120k EV surface, we performed immunogold TEM. Gold particles indicating antibody binding to *Ov*-TSP-2 (**Figure 1B**) and *Ov*-TSP-3 (**Figure 1C**) was observed on the surface of 120k EVs probed with anti-TSP but not negative control sera (**Figure 1D**). Moreover, equal volumes of fractions F1-F12 following iodixanol separation were loaded on SDS-PAGE gels for western blot analysis to determine the distribution of *Ov*-TSP-2 and *Ov*-TSP-3 among the fractions. *Ov*-TSP-2 and *Ov*-TSP-3 were both observed in the ultracentrifugation pellet (before iodixanol gradient) and were predominantly observed in F7 (density of 1.105 g/ml) after iodixanol gradient. While *Ov*-TSP-2 was detected in F5-F10 (density 1.084-1.239 g/ml), *Ov*-TSP-3 was detected in F6-F10 (1.097-1.239 g/ml) (**Figure 1E**). These proteins were not present in other fractions containing contaminant soluble proteins and other vesicles.

### *O. viverrini* 120k EV Proteome

**Figure 2:**
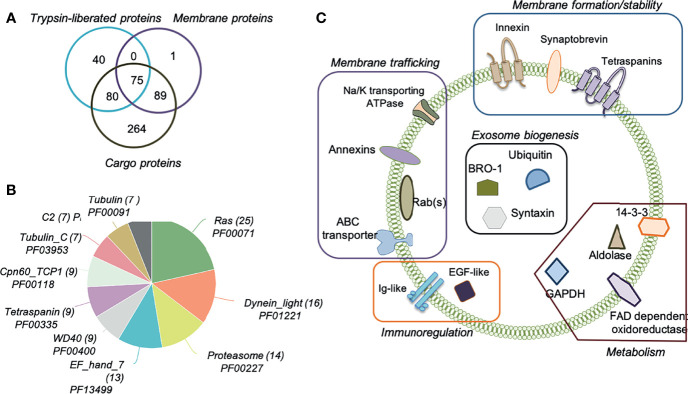
Proteomic analysis of *Opisthorchis viverrini* 120k extracellular vesicle sub-vesicular compartments. Extracellular vesicles (EVs) were prepared using an Optiprep iodixanol gradient and then subjected to sequential extraction and proteomics analysis to characterise the number of EV proteins identified by (i) enzymatic shaving (Trypsin-liberated proteins); (ii) membrane proteins remaining after enzymatic shaving, and (iii) intra-vesicular cargo proteins **(A)**. Most represented Pfam protein families and domains in the identified proteins were determined **(B)**. Schematic representation of important proteins present on the surface, anchored in the membrane and within the cargo of *O. viverrini* 120k EVs **(C)**.

Highly pure *O. viverrini* 120k EVs obtained after iodixanol gradient were subjected to a proteomic analysis. To maximise the number of identifications and localise the proteins within the different fractions of the EVs, we “shaved” exposed vesicle surface proteins using a mild trypsin digestion and, subsequently, cargo and membrane proteins were characterised. We confidently identify 549 unique proteins with 2 or more unique peptides, including 195 as trypsin-liberated (or “shaved”) proteins (**Supplementary Table S3**), 165 as membrane proteins (**Supplementary Table S4**) and 505 as cargo proteins (**Figure 2A** and **Supplementary Table S5**). A Pfam analysis of the families and domains present in these 549 proteins revealed that the Ras family (PF00071) was most highly represented, with 25 proteins belonging to this family, followed by Dynein light chain (PF01221, 16 proteins) and Proteasome (PF00227, 14 proteins) (**Figure 2B**). Not surprisingly, 9 proteins belonged to the Tetraspanin family (PF00335), including Uroplakin (OON13593.1, OON14078.1 and OON19878.1), CD63 (OON14177.1, OON14450.1, OON15235.1, OON16191.1 and OON16870.1) and CD9/81 (OON15358.1) groups. Two of these sequences (OON16870.1 and OON14450.1) belong to the CD63 family of tetraspanins and had already been identified by our group (Chaiyadet et al., 2017) (**Supplementary Table S6**). Several proteins involved in exosome biogenesis (such as BRO-1, ubiquitin and syntaxin), as well as in immunoregulation (Ig-like and EGF-like proteins), metabolism (GAPDH, 14-3-3, aldolase, FAD dependent oxidoreductase among others) and membrane trafficking (annexins, ABC transporter and Na/K transporting ATPase among others) were also identified (**Figure 2C**). In addition to tetraspanins, other molecules involved in membrane formation and stability were identified (i.e. innexin and synaptobrevin).

To gain insight into the processes and functions mediated by the proteins present in the *O. viverrini* 120k EVs, a gene ontology analysis was performed. Several metabolic as well as red-ox, transportation and proteolytic processes can be regulated by these proteins (**Supplementary Figure S1A**). Protein-, metal ion- and ATP-binding molecular functions were afforded the highest nodescores by Blast2GO (**Supplementary Figure S1B**). In addition, other functions such as transferase, nucleoside-triphosphatase and catalytic activity were also predicted to be regulated by *O. viverrini* 120k EVs proteins. As expected, most of the proteins belonged to the cellular compartments “integral component of membrane” and “cytoplasm” (**Supplementary Figure S1C**).

### Inhibition of EV Internalization by Endocytosis Pathway Inhibitors

**Figure 3:**
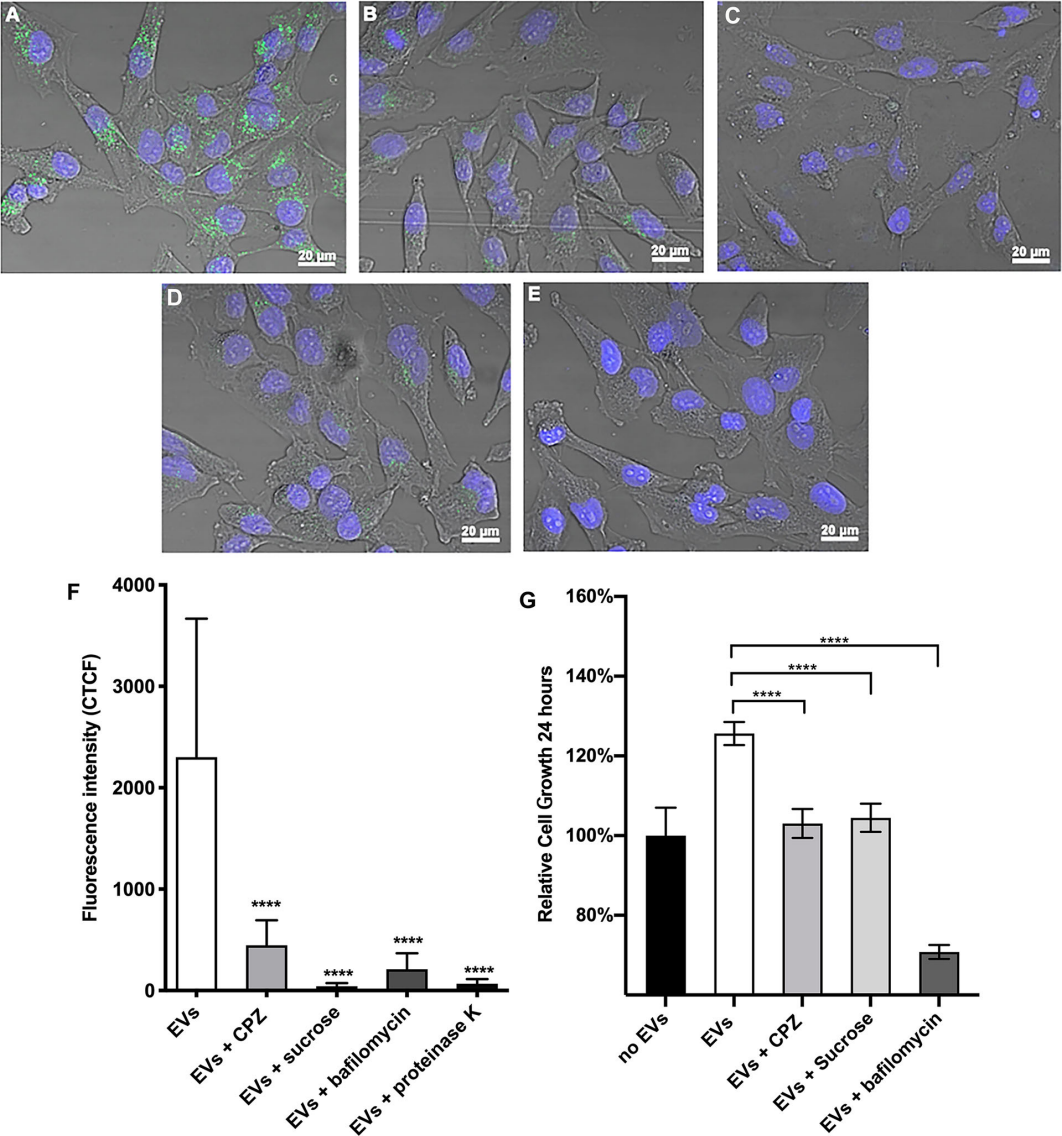
Chemical inhibition of endocytosis pathways blocks the uptake of *O. viverrini* 120k extracellular vesicles and reduces proliferation of cholangiocytes. H69 human cholangiocytes were cultured with PKH67-labeled *O. viverrini* 120k extracellular vesicles (EVs) for 2 hours in the presence of PBS control **(A)**, chlorpromazine (CPZ) **(B)**, sucrose **(C)** or bafilomycin A1 **(D)**. PKH67-labeled 120k EVs were also pre-treated with proteinase K prior to co-culture with H69 cholangiocytes **(E)**. Corrected total cell fluorescence was quantified by imageJ **(F)**. Cholangiocyte proliferation induced by 120k EVs in the presence or absence of endocytosis inhibitors was measured in real time using an xCELLigence system **(G)**. Thirty cells were counted form 3 random microscope fields for each sample in 2 independent experiments. Values are mean ± SD. The corresponding p-values (****P < 0.0001) were performed by one-way ANOVA with Tukey-Kramer *post-hoc* multiple comparison test.

To investigate the pathway(s) implicated in the internalization of *O. viverrini* 120k EVs by H69 cholangiocytes, different endocytosis inhibitors (sucrose, CPZ and bafilomycin A1) were employed. Cells were pretreated with inhibitors prior to incubation with PKH67-labelled *O*. *viverrini* 120k EVs. Pretreatment of H69 cells with inhibitors for clathrin-mediated endocytosis such as CPZ and sucrose resulted in 80% and 98% reduction of EVs uptake compared to control (untreated) cells (**Figures 3A–C**). Bafilomycin A1, a non-clathrin mediated inhibitor also significantly blocked (90% reduction) uptake of EVs by H69 cells (**Figure 3D**). Furthermore, pretreatment of *O*. *viverrini* 120k EVs with proteinase K reduced cellular uptake by 97% compared to controls (**Figure 3E**). Corrected total cell fluorescence (CTCF) was determined and statistical analyses performed (**Figure 3F**). These results suggest that *O. viverrini* 120k EVs can be internalized by H69 cholangiocytes by various endocytic mechanisms, including clathrin and non-clathrin-mediated pathways.

To determine whether endocytosis of PKH67-labelled *O. viverrini* 120k EVs by cholangiocytes promoted cell proliferation we monitored cell growth in real-time using an xCelligence system (Xing et al., 2005). H69 cholangiocytes were pre-treated with the endocytosis inhibitors CPZ, sucrose and bafilomycin A1 for 30 minutes before culturing with *O. viverrini* 120k EVs and then monitored for 24 h. All of the endocytosis inhibitors tested significantly reduced the ability of *O*. *viverrini* 120k EVs to stimulate cholangiocyte proliferation at 24 h (**Figure 3G**).

### Antibodies against *O. viverrini* Tetraspanins Block EV Internalization

**Figure 4:**
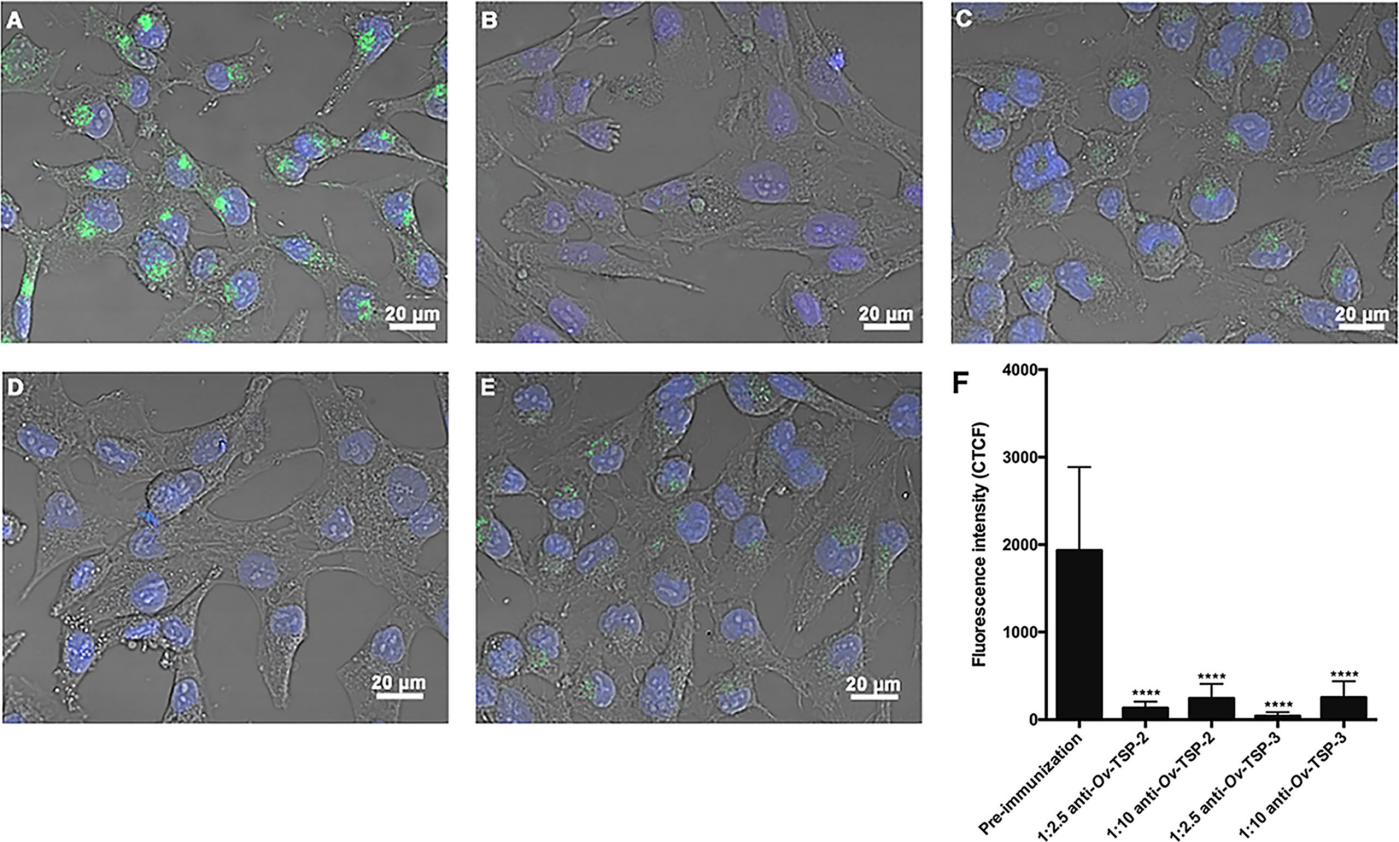
Antisera against recombinant *Ov*-TSP-2 and *Ov*-TSP-3 blocked the uptake of *O. viverrini* 120k extracellular vesicles by cholangiocytes. H69 human cholangiocytes were cultured with PKH67-labeled *O*. *viverrini* 120k extracellular vesicles (EVs) in the presence of rabbit pre-immunization serum **(A)**, rabbit antiserum to recombinant *Ov*-TSP-2 at dilutions of 1:2.5 and 1:10 **(B, C)**, rabbit antiserum to recombinant *Ov*-TSP-3 at dilutions of 1:2.5 and 1:10 **(D, E)**. Corrected total cell fluorescence was measured at 490 nm (green channel) using ImageJ **(F)**. The nuclei were stained by Hoechst (blue color). Thirty cells were counted form 3 random microscope fields for each sample in 2 independent experiments. Values are mean ± SD. The corresponding p-values (****P < 0.0001) were obtained by one-way ANOVA with Tukey-Kramer *post-hoc* multiple comparison test.

*Ov*-TSP-2 and TSP-3 belong to the CD63 family of tetraspanins. The CD63 family has been identified as a biomarker of exosomes from mammalian cells. Rabbit antisera against *Ov*-TSP-2 and *Ov*-TSP-3 or pre-immunization control sera were pre-incubated with *O. viverrini* PKH67-labelled 120k EVs prior to their addition to cholangiocytes. Antisera to recombinant *O. viverrini* TSP-2 and TSP-3 reduced EVs internalization by cholangiocytes by 93% and 97% respectively (*P*<0.0001, **Figures 4B, D, F**), when compared to pre-immunization serum (**Figures 4A, F**).

### Silencing of Ov-tsp-2 and tsp-3 Gene Expression Reduces EV Secretion and Attenuates Ov-TSP Protein Expression

**Figure 5:**
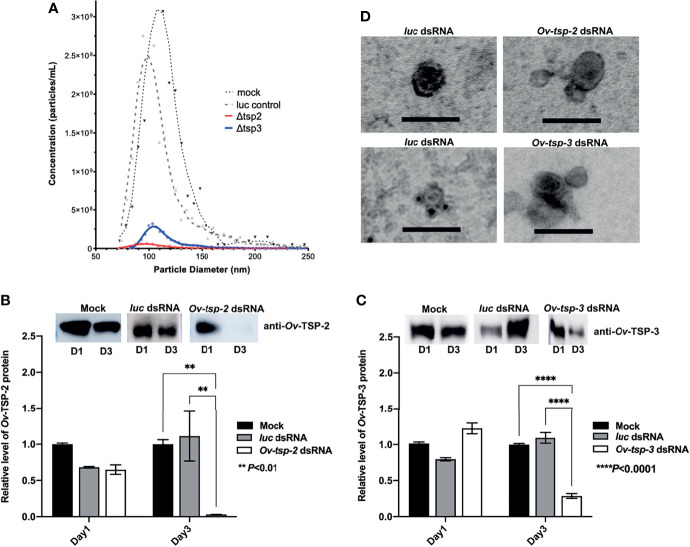
RNA interference-mediated silencing of *Ov-tsp-2* and *Ov*-*tsp-3* substantially reduced 120k extracellular vesicle secretion by adult *Opisthorchis viverrini* in culture. *O. viverrini* 120k extracellular vesicles (EVs) were purified from excretory/secretory products of flukes treated for one (D1) or three (D3) days with *Ov-tsp-2*, *Ov*-*tsp-3* or firefly luciferase dsRNAs *in vitro*. EVs collected on D3 were quantified by tunable resistive pulse sensing using a qNano instrument **(A)**. EVs from dsRNA-treated worms were probed with antisera to *Ov*-TSP-2 **(B)** and *Ov*-TSP-3 **(C)** by western blot and band intensity was determined using ImageJ software. Confirmation of the absence of *Ov*-TSP-2 and TSP-3 in EVs from dsRNA-treated flukes was performed using immunogold transmission electron microscopy **(D)**. Black dots (antibody binding) are visible on EVs from *luc* dsRNA-treated flukes but not EVs from *Ov-tsp-2* or *Ov-tsp-3* dsRNA-treated flukes when probed with homologous antisera. The black scale bar denotes 100 nm.

To determine whether silencing of the *Ov-tsp-2* and *tsp-3* genes affected EV secretion, we collected 120k EVs from culture supernatant after three days and quantified them by TRPS. Silencing of either *tsp* gene did not substantially impact on the mean diameter of the secreted vesicles (104-116 nm) but did result in a substantial reduction in the number of vesicles secreted by *tsp-2* (4.17×10^8^/ml) and *tsp-3* (25.2×10^8^/ml) dsRNA-treated flukes compared to both *luc* (2×10^10^/ml) and mock (2.29×10^10^/ml) controls (**Figure 5A**). To confirm the presence or absence of the respective TSPs in 120k EVs from dsRNA-treated worms, 2 µg of EV total protein (8×10^7^ vesicles) per lane was electrophoresed by SDS-PAGE and transferred to nitrocellulose membrane then probed with anti-*Ov*-TSP-2 and *Ov*-TSP-3 rabbit sera and detected by chemiluminescence. Silencing of *Ov-tsp-2* expression over a 3 day period resulted in 97% reduction (** *P*< 0.01) in TSP-2 protein levels in EVs when compared to mock control and *luc* dsRNA-treated EVs (**Figure 5B**), and absence of the TSP-2 protein by immunogold TEM (**Figure 5D**). Silencing of *Ov-tsp-3* expression resulted in 72% reduction (**** *P*<0.0001) in TSP-3 protein levels in EVs when compared when compared to mock control and *luc* dsRNA-treated EVs (**Figure 5C**), and absence of the TSP-3 protein by immunogold TEM (**Figure 5D**). Both proteins were readily detectable in *luc* dsRNA-treated flukes using both western blot (**Figures 5B, C**) and immunogold TEM (**Figure 5D**).

## Discussion

The roles that EVs play in inter-phylum molecular communication is becoming readily apparent, notably for the promise they hold in developing next-generation therapeutics (Woith et al., 2019). The protection conferred by the double lipid bilayer to the molecular cargo present in EVs (i.e. proteins, glycans, miRNAs and small molecules), as well as the ability of EVs to securely deliver their payloads to distant tissues around the body confers a distinct advantage over soluble pathogen mediators that likely act more locally. The proteomic and genomic cargo of several helminths has already been analysed (Buck et al., 2014; Sotillo et al., 2016; de la Torre-Escudero et al., 2019; Hansen et al., 2019), however, the mechanisms involved in helminth-derived EV internalization is poorly understood. Addressing the processes of helminth EV uptake by mammalian host cells may aid in the development of effective strategies to detect and destroy helminths such as *O. viverrini*. Herein we provide an in-depth characterization of the proteomic content of *O. viverrini* 120k EVs and investigated the pathways and key molecules involved in their production and uptake.

Several methods have been used to isolate and purify EVs from helminths, although most studies utilized just ultracentrifugation, including our own previous report on *O. viverrini* EVs. However, ultracentrifugation alone is now considered insufficient as per the MISEV guidelines (Thery et al., 2018), since it might not fully remove small molecules and soluble protein contaminants. To achieve a highly pure EV preparation, we isolated EVs using an Optiprep density gradient and compared it to EVs isolated by ultracentrifugation alone. The number of EVs isolated using both methods was comparable, however the EV preparation using Optiprep was of greater purity, confirmed by the presence of exosome markers such as TSPs in several of the fractions obtained. We recommend that future studies focusing on fluke 120k EVs use Optiprep density gradient or other comparable isolation techniques to obtain sufficiently pure samples, particularly when performing biological studies.

To gain insights into the molecules transported within the EVs and the proteins potentially involved in EV-cell interactions, we followed established methods to isolate the proteins present in the membrane and within the cargo of EVs (Cwiklinski et al., 2015). Similar to what has been observed in other liver flukes such as *F. hepatica*, several proteins involved in the ESCRT-dependent exosome biogenesis pathway (i.e. BRO-1, ubiquitin and syntaxin), as well as SNARE proteins involved in vesicle trafficking and fusion (i.e. synaptobrevin) were found. There are currently no EV biomarkers for liver flukes (and helminths in general) (Sotillo et al., 2020); however, our results suggest that the population of *O. viverrini* 120k EVs isolated after Optiprep separation likely correspond to the population of EVs that have previously been referred to from other organisms (mostly mammals) as exosomes. We also confirmed the presence of metabolic enzymes (i.e. aldolase, GAPDH, 14-3-3), proteins involved in membrane trafficking (annexins, ABC transporter) and several tetraspanins identified in EVs secreted by *O. viverrini* and other helminths (Chaiyadet et al., 2015b; Cwiklinski et al., 2015; Eichenberger et al., 2018; Kifle et al., 2020b).

Several mechanisms have been described for the uptake of EVs and release of their content into recipient cells, including clathrin-mediated (CME), and independent endocytosis (CIM). CME requires a ligand and receptor for interaction and internalization into recipient cells (Conner and Schmid, 2003). Chlorpromazine has been described as a potent CME inhibitor by inhibiting the clathrin protein retrieved to the plasma membrane and, thus, blocking the endocytosis process (Khalil et al., 2006). Chemical inhibition of the CME pathway blocked internalization of *O. viverrini* 120k EVs and attenuated EV-induced proliferation of cholangiocytes. Likewise, EV uptake by cholangiocytes was blocked by sucrose, which forms a hypertonic milieu inducing abnormal clathrin polymerization (Heuser and Anderson, 1989). We also confirmed endocytosis inhibition using bafilomycin A1, a vacuolar H+ATPase pump inhibitor that is necessary for receptor-mediated endocytosis (Harada et al., 1996), suggesting that *O. viverrini* 120k EVs can also be internalized by cholangiocytes through micropinocytosis. These findings agree with previously published studies showing that CME and CIM pathways are involved in the internalization of *O viverrini* crude ES products (Chaiyadet et al., 2015a). In the case of *O. viverrini* 120k EVs, the involvement of different endocytic pathways in cellular uptake might be explained by different surface components of EVs and specific receptors of distinct target cells that can impact their uptake. Furthermore, as shown in the EV surface proteome, *O. viverrini* EVs contain dozens of surface proteins, many of which might act as ligands for receptors on host cells, facilitating a multi-factorial recognition and uptake process. In like fashion, *S. mansoni* EVs contain N-glycans with LewisX motifs at the surface which serve as a ligand for DC-SIGN on human monocyte-derived dendritic cells (Kuipers et al., 2020), but whether this reflects specific and targeted uptake of EVs or MHC-mediated processing by professional antigen presenting cells is unclear. Removal of the surface proteome of *O. viverrini* 120k EVs with proteinase K prevented internalization of EVs by cholangiocytes, suggesting an essential role for one or more proteins on the surface membrane.

Tetraspanins belonging to the CD63 family have been shown to be exosome markers in both mammalian cells and in other parasitic organisms such as the related liver fluke *Fasciola hepatica* (Andreu and Yanez-Mo, 2014; Ramos et al., 2016; de la Torre-Escudero et al., 2019), and were shown in earlier studies by us (Chaiyadet et al., 2015b) and herein to be abundantly represented in *O. viverrini* EVs. We suggested a role for TSPs in the maintenance of tegumental structure in *O. viverrini*, since silencing of *Ov-tsp*-2 and *Ov-tsp-3* using RNA interference resulted in the loss of integrity of the tegument (Chaiyadet et al., 2017). Surprisingly, despite tegument malformation in flukes treated with *Ov-tsp*-2 and *Ov-tsp-3* dsRNAs, EVs were still released *in vitro* and detected in the ES products by TRPS and TEM. Unfortunately, the reduced numbers of 120k EVs secreted by flukes that were treated with *Ov-tsp* dsRNAs were insufficient to undertake comprehensive host cell uptake studies, however, antibodies against these recombinant tetraspanins have been convincingly shown by us to prevent EV uptake by cholangiocytes, supporting an essential role for *Ov*-TSP-2 in the internalisation process. We cannot unequivocally conclude that *Ov*-TSP-2 directly interacts with a host cell receptor, and its knock down might instead result in destabilization of the EV membrane tetraspanin web (Rana et al., 2012), thereby affecting the general surface architecture and integrity of the vesicles and their subsequent interactions with target host cells. Similarly, tetraspanins were recently shown to be important for internalization of *S. mansoni* EVs into mammalian host target cells (Kifle et al., 2020a).

In mice where the *CD9* TSP gene was knocked out, exosome secretion was shown to be defective in bone marrow dendritic cells in comparison to wild type counterparts (Chairoungdua et al., 2010). On the other hand, gene editing of *CD63* (the family of TSPs to which *Ov*-TSP-2 and TSP-3 belong) using CRISPR/Cas9 knockout in HEK293 cells resulted in a decrease in exosome (but not larger MV) secretion while cell viability and doubling time remained unchanged, suggesting the importance of CD63 in exosome biogenesis (Hurwitz et al., 2016).

Silencing of *Ov-tsp-2* and *tsp-3* gene expression using RNAi did not completely ablate EV secretion by day 3 but substantially reduced the number of vesicles secreted. Furthermore, those vesicles that were secreted had significantly reduced levels of their respective TSP proteins targeted by RNAi. In earlier work we showed that silencing of *Ov-tsp-2* gene expression in adult flukes resulted in a major malformation of the parasite tegument (Chaiyadet et al., 2017), a finding that might account for the reduction in the number of EVs secreted herein.

CD63 in mice has been shown to directly participate in ESCRT-independent sorting but not of traditional ESCRT-dependent cargos to intraluminal vesicles, thereby participating in the biogenesis of lysosome-related organelles (van Niel et al., 2011), although EV secretion was not assessed in this study. CRISPR/Cas9 knockout of CD63 in Epstein-Barr virus infected cancer cells resulted in reduced secretion of EVs that were positive for the viral protein LMP1, and severely impaired packaging of those vesicles, concomitant with a disruption in the perinuclear localization of LMP1 (Hurwitz et al., 2017). The CD9 tetraspanin is involved in cell fusion, adhesion and motility, and anti-human CD9 Fab fragment binds to CD9 on both EVs and target cells (Santos et al., 2019). Silencing of the *CD9* gene both in EVs and recipient cells decreased EV endocytosis by target cells and abolished the nuclear transfer of EV cargo (Rappa et al., 2017). Moreover, monovalent anti-CD9 Fab fragments reduced EV uptake and the nuclear transfer of their proteins in all examined cell lines (Santos et al., 2019). Although we could not assess the cellular uptake of fluke EVs where *tsp* genes were silenced, anti-TSP antibody-mediated approaches can interrupt EV uptake by target cells, thereby supporting a key role for TSPs in the internalization process. However, cellular uptake studies with EVs where *tsp* gene expression is knocked down or knocked out need to be performed to definitely address the role of TSPs in this process.

TSPs from parasitic helminth EVs are showing great promise as efficacious subunit vaccines against helminth infections (Mekonnen et al., 2018; Drurey et al., 2020). Indeed, *Ov*-TSP-2 was recently shown to induce protective IgG and IgA responses after oral delivery of recombinant *Bacillus subtilis* spores expressing the TSP-2 LEL fragment to hamsters followed by challenge infection (Phumrattanaprapin et al., 2021). Human cholangiocytes that internalize *O. viverrrini* EVs undergo rapid proliferation and secrete inflammatory cytokines, all of which contribute to the development of bile duct cancer in naturally infected humans (Sripa et al., 2007) and experimentally infected hamsters (Flavell and Lucas, 1983), so it is likely that an ultimate subunit vaccine that blocks uptake of parasite EVs by bile duct cells of infected individuals will have both anti-infective and anti-cancer properties, not unlike the human papillomavirus vaccine.

In summary, we show here that the CD63 family TSPs on the surface of EVs secreted by the parasitic liver fluke *O. viverrini* are potential biomarkers of 120k EVs, and that *tsp* gene silencing ablates tetraspanin protein expression at the EV surface and reduces the rate of EV secretion by flukes *in vitro*. Our findings support an essential role for tetraspanins in fluke EV biogenesis and/or secretion, and shed light on the mechanisms by which these proteins are efficacious vaccine antigens.

## Data Availability Statement

The datasets presented in this study can be found in online repositories. The names of the repository/repositories and accession number(s) can be found in the article/**Supplementary Material**.

## Ethics Statement

The animal study was reviewed and approved by Animal Ethics Committee of Khon Kaen University (ACUC KKU 31/2561).

## Author Contributions

SC, JS, PB, TL, and AL conceived the project and designed the experiments. SC, JS, ST, MS, and WK performed the experiments and analyzed the results. All authors reviewed the manuscript.

## Funding

This study was supported by the National Cancer Institute, National Institutes of Health, award no. 2R01CA164719-06A1 (TL, AL and PB) and Research and Graduate Studies, Khon Kaen University (TL and SC). AL is supported by a senior principal research fellowship (1117504) and program grant (1132975) from the National Health and Medical Research Council, Australia. The Transmission Microscopy facility at Khon Kaen University was supported by the Department of Anatomy, Faculty of Medicine, Khon Kaen University.

## Conflict of Interest

The authors declare that the research was conducted in the absence of any commercial or financial relationships that could be construed as a potential conflict of interest.

## Publisher’s Note

All claims expressed in this article are solely those of the authors and do not necessarily represent those of their affiliated organizations, or those of the publisher, the editors and the reviewers. Any product that may be evaluated in this article, or claim that may be made by its manufacturer, is not guaranteed or endorsed by the publisher.

## Supplementary Material

Supplementary Figure 1 Gene ontology analysis of proteins present in *Opisthorchis viverrini* 120k extracellular vesicles. Biological processes **(A)**, molecular functions **(B)** and cellular components **(C)** of the proteins present in *O. viverrini* 120k extracellular vesicles. Colour represents nodescore from Blast2GO, while size represents the number of sequences containing that particular gene ontology.

Supplementary Table 2 Characterization of the density, size, number and purity of *Opisthorchis viverrini* 120k extracellular vesicles obtained after ultracentrifugation or iodixanol gradient.

Supplementary Table 6 Percent sequence identity between *O. viverrini* CD63-like tetraspanins, *Ov*-TSP-2 and TSP-3 with their closest human and hamster host homologues.
